# Review of "The blood-brain and other neural barriers reviews and protocols" by Sukriti Nag (Editor)

**DOI:** 10.1186/2045-8118-8-17

**Published:** 2011-05-08

**Authors:** David J Begley

**Affiliations:** 1 Institute of Pharmaceutical Science, Kings College London, Hodgkin Building, Guys Campus, London SE1 1UL, UK

## Abstract

This is a review of the content and scope of a multi-author volume for readers with an interest in the structure and function of the blood-brain barrier and in drug delivery to the central nervous system.

## 

This volume is one of a number in the Methods in Molecular Biology series Volume 686. It is designed as a sequel to "The Blood-Brain Barrier - Biology and Research protocols which appeared under the same publishers as part of the Methods in Molecular Medicine Volume 89 (2003). The book follows a slightly different structure to previous volumes in that it contains an number of reviews bringing key areas of the subject, which have shown significant recent development, up to date, plus further sections which contain specific protocols for blood-brain barrier study and drug delivery to the central nervous system. The volume is divided into five parts, I "Biology of the Barrier", II "Imaging the Barrier", III "Molecular Techniques to Study the Blood-Brain Barrier", IV "Models to Study the Barrier" and V "Delivery of Therapeutic Agents Across the Barrier".

Part I contains six reviews which bring critical areas up to date. There is a chapter on endothelia cells (Nag), pericytes (Dore-Duffy and Cleary), astrocytes (Nag), the blood-cerebrospinal fluid barrier (Johanson, Stopa and McMillan), the blood-retinal barrier (Runkle and Antonetti) and finally the blood-nerve barrier (Weerasuriya and Mizisin). In this way the opening section reviews the morphology and function of the cell associations forming the barriers of the CNS and directly reviews current knowledge of the different barriers between the nervous system and the systemic circulation.

Part II is devoted to imaging the barrier and covers confocal microscopical detection of proteins in endothelial cells (Manias, Kapadia and Nag), MRI of permeability of contrast agents (Nagaraja et al.), pathological investigations with iron oxide microparticles (Anthony et al.), human blood-brain barrier integrity measured with MRI (Kassner and Thornhill), the blood cerebrospinal fluid barrier in the embryonic rat (Saunders et al.) and MRI studies of the blood-nerve barrier with MRI (Wessig).

Part III moves on to molecular techniques and covers protocols for isolation of endothelia cells and a study of lipid rafts with mass spectroscopy (Cayrol et al), Laser capture proteomic studies of endothetlial cells, (Murugesan), P-glycoportein expression and function (Chan and Bendayan), and methods to study glycoproteins in the blood-brain barrier using mass spectroscopy (Haqqani et al.).

Part IV covers models available for barrier study including novel systems for the eye and brain in Drosophila (Pinsonneault et al.) and zebrafish brain (Eliceiri, Gonzalez and Baird). Cell culture models for pericyte endothelial interactions in a porcine model (Thanabalassundaram et al.), the human outer retinal barrier (Hamilton and Leach), and the peripheral nerve barrier (Sano and Kanda) are then described and discussed.

The final part V is devoted to the delivery of therapeutic agents across the barrier and includes, treatment of focal ischemia with viral vector mediated gene transfer (Su and Yang), Treatment of brain tumours with barrier disruption (Blanchette and Fortin), the use of liposome nanocarriers and single domain antibodies for delivery of drugs and contrast agents in the context of drug delivery (Iqbal, Abulrob and Stanimirovich) and finally chapter on targeting the choroid plexus and cerebrospinal fluid using peptide motifs identified by page display (Baird et al.)

In all this book represents a very useful addition to the series, the reviews and protocols bring important areas within the field up to date in a very accessible format and will be attractive to both established scientists in the area and postgraduate and postdoctoral students establishing a career and wishing to master new ideas and methods. It should be on the bookshelf of every lab with an interest in the blood-brain barrier. Again Sukriti Nag has brought together a panel of world experts to compile a valuable volume.

## Competing interests

The author declares that they have no competing interests.

## Authors' contributions

Sole author.

